# Detection and Profiling of Antibiotic Resistance among Culturable Bacterial Isolates in Vended Food and Soil Samples

**DOI:** 10.1155/2020/6572693

**Published:** 2020-09-04

**Authors:** Susan W. Muriuki, Johnstone O. Neondo, Nancy L. M. Budambula

**Affiliations:** ^1^Department of Biological Sciences, University of Embu, P.O. Box 6-60100, Embu, Kenya; ^2^Institute for Biotechnology Research, Jomo Kenyatta University of Agriculture and Technology, P.O. Box 62000-00200, Nairobi, Kenya

## Abstract

The emergence and persistence of antibiotic resistance remain formidable health challenges. This study aimed at detecting and profiling antibiotic resistance of bacterial contaminants in vended food and the environment. Seventy antibiotic-resistant bacterial isolates were isolated from fried fish, African sausages, roasted meat, smokies, samosa, chips (potato fries), vegetable salads, and soil samples collected from Embu Town and Kangaru Market in Embu County, Kenya. The antibiotic susceptibility test, morphological and biochemical characterization, antibiosis assay, polymerase chain reaction-based detection of antibiotic resistance genes, and sequencing of the 16S rRNA gene were done. Analysis of variance on all measured data was done, and Tukey's honest test was used to compare and separate mean diameters of zones inhibition. Resistance of bacterial isolates to antibiotics was chloramphenicol (90%), cefotaxime (84.29%), nalidixic acid (81.43%), tetracycline (77.14%), amoxicillin (72.86%), gentamycin (48.57%), streptomycin (32.86%), and trimethoprim + sulphamethoxazole (30%). Isolate KMP337, *Salmonella* spp., exhibited highly significant antibiosis against *S. aureus* recording a mean inhibition diameter and standard error (SE) of 16.33 ± 0.88 mm, respectively, at *P*=0.001. The 70 bacterial isolates belonged to *Bacillus*, *Paraclostridium*, *Lysinibacillus*, *Virgibacillus,* and *Serratia* genera. The study isolated *Bacillus wiedmannii* (KC75) which is a risk group 2 as well as *Serratia marcescens* (KMP95) and *Bacillus anthracis* (KS606) which are risk group 3 organisms. The presence of antibiotic resistance genes *Tet* A, ^*Bla*^*TEM, StrB, Dfr* A*, Amp,* and *FloR* genes was confirmed by a polymerase chain reaction. Samples from Kangaru Market recorded a higher (88.57%) proportion of resistant isolates as compared to isolates from Embu Town (11.43%). The study confirmed the presence of antibiotic-resistant bacteria in vended fast food and the soil in Embu Town and Kangaru Market. This study calls for continuous monitoring of bacterial status and hygienic handling of vended food.

## 1. Introduction

Urban centers in developing countries face socioeconomic challenges that can adversely affect health standards. Foodborne diseases, particularly those of microbial origin, are a major health problem and contribute to reduced economic growth [1]. Street foods are popular in urban areas because of their availability, convenience, and affordability. However, the hygiene aspects of sales operations are a major source of concern for the health of both handlers and consumers [2]. Running surface water, inadequate sanitation, and the disposal of untreated wastewater provide an environment conducive to the growth of pathogenic microbes. Besides, street foods are prone to cross-contamination due to lack of personal hygiene, sharing of contaminated utensils, and free movement of flies that sporadically land on the food.

Food and environmental samples are reservoirs of foodborne pathogens such as *Listeria monocytogenes, Vibrio parahaemolyticus, Escherichia coli, Salmonella* spp.*, Vibrio cholerae*, and *Staphylococcus aureus* [1, 3]. In addition to antibiotic resistance in bacterial pathogens, commensal bacteria and the environment are also reservoirs of antibiotic resistance. The release of animal and human waste into the environment results in the subsequent release of antibiotics into water and soil, which encourages further spread of antibiotic resistance [4]. Bacterial resistance to commonly used antibiotics has been associated with enteric pathogens such as *E. coli, Salmonella* spp*., Shigella* spp*., Klebsiella* spp., and *V. cholerae* [5].

A cross-sectional study conducted in 2014–2016 in 22 of Kenya's 47 counties diagnosed 11,033 people with cholera [6]. An outbreak of cholera was reported in Embu County in 2019 [7]. Waterborne diseases in Embu County have been attributed to poor sanitation and contamination of water sources [8]. Despite efforts to report the prevalence of food- and waterborne pathogens in Embu County, data on their susceptibility to antibiotics are scarce, so this study focused on the isolation and characterization of antibiotic-resistant bacteria in food sold in Embu Town and the nearby Kangaru Market.

## 2. Materials and Methods

### 2.1. Study Site and Sampling

**Figure 1:**
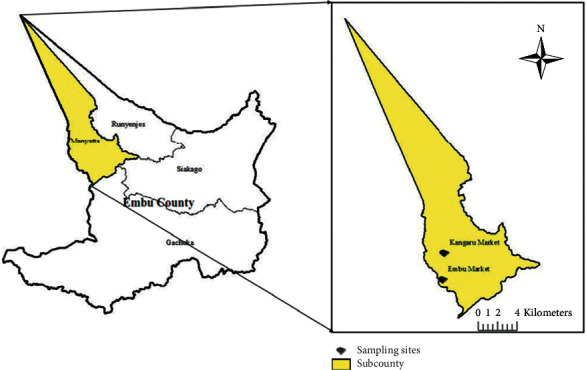
Sampling site in Embu County.

Samples consisting of fried fish, African sausages, roasted meat, smokies, samosa, chips (potato fries), vegetable salads, and soil were purposively collected from 176 out of the 324 licensed food vendors in the densely populated Central Business District of Embu Town and the nearby Kangaru Market (latitude: 0°31′58.80″N and longitude: 37°27′0.00″) (Figure 1). Samples were collected aseptically and transported to the Microbiology Laboratory at the University of Embu where they were stored in a refrigerator at 4°C. Treated water from Embu Water and Sanitation Company (EWASCO) was also sampled.

### 2.2. Preparation of Inoculum and Subculturing

The samples were incubated in eight selective media, namely, MacConkey agar, Salmonella Shigella agar, brain-heart infusion agar, phenylalanine agar, Pseudomonas fluorescens agar, brilliant green agar, eosin ethidium bromide agar, and deoxycholate agar. The standard cultivation method for bacterial food contaminants isolation was carried out as recommended by the International Organization for Standardization (ISO) 6579 [9]. The plates were incubated at 37°C for 24 hours, in an inverted position, after which single colonies were subcultured on fresh media.

### 2.3. Characterization and Identification of Bacterial Isolates

The isolates were characterized as described in [10]. The ability of the isolates to ferment sugars, utilize citrate, and produce catalase enzyme and phenyl-pyruvic acid was determined as previously described [11, 12]. The isolates were screened for their ability to produce amylase, protease, cellulase, and esterase using the methods described in [13, 14]. Probable identification of the isolates was done using Bergey's manual of determinative bacteriology and confirmed by partial sequencing of the 16S rRNA gene of some isolates.

### 2.4. Antibiotic Susceptibility Assay

The Kirby Bauer disc diffusion method using Mueller-Hinton agar was used for antimicrobial susceptibility testing [15]. Antimicrobial discs with amoxicillin (20 *µ*g), cefotaxime (30 *µ*g), gentamicin (10 *µ*g), streptomycin (10 *µ*g), tetracycline (30 *µ*g), nalidixic acid (30 *µ*g), trimethoprim + sulphamethoxazole (1.25/23.75 *µ*g), and chloramphenicol (30 *µ*g) were used. *Klebsiella pneumoniae* 1792880 and *E. coli* 35218 were used as quality control strains. Bacterial suspensions of pure isolates were prepared based on 0.5 McFarland Turbidity Standard. The plates were incubated in an inverted position at 37°C for 24 hrs. The diameter of the zones of inhibition (in millimeters) was measured and interpreted as either susceptible, intermediate, or resistant according to the Guidelines of CLSI [16].

### 2.5. Antibiosis Test

Standardized bacterial suspensions were prepared based on 0.5 McFarland turbidity [17]. The antibiosis test was performed as previously described [18]. The isolates were incubated at 37°C and monitored routinely after every 24 hours for two days. The diameter of the zones of inhibition was recorded. The antibiotic-resistant bacteria were tested for antibiosis against six laboratory bacteria strains, namely, *Staphylococcus aureus* ATCC 1026, *Klebsiella pyrope* 1792880, *E. coli* ATCC 35218, *Streptococcus pneumoniae* ATCC 49619, *S. aureus* BAA 976, and *K. pneumoniae* 1792880.

### 2.6. DNA Extraction from Antibiotic-Resistant Bacteria

The bacterial isolates were grown overnight in Luria Broth for 24 hours, and DNA was extracted using the phenol-chloroform method with some modifications [19]. To isolate plasmid DNA, bacterial isolates were grown overnight in Luria Broth. Plasmids were extracted using a protocol modified from [20].

### 2.7. Partial Sequencing of Bacterial 16S rRNA Gene

The 16S rRNA gene sequence was amplified by PCR using bacterial universal primers 8F (5′-AGCTTTGATCCTGGCTCAG-3′) and 1492R (5′-CGGCTACCTTGTTACGACTT-3′) for all selected bacterial isolates according to [21] using Phusion-High fidelity PCR Kit (New England and Biolabs Inc). The PCR amplicons were checked by gel electrophoresis, labeled, and shipped to Inqaba Biotech African's Genomics Company in South Africa for sequencing. The sequence outputs were edited, analyzed, and deposited in GenBank for assignment of accession numbers.

### 2.8. PCR Amplification of Antibiotic Resistance Genes

Genes encoding resistance to tetracycline, streptomycin, and beta-lactam antibiotics such as ampicillin, chloramphenicol, and trimethoprim were detected using the primer specific PCR approach (Table 1) as described in [22]. The presence of a target gene was confirmed by the presence of a band on the gel.

### 2.9. Data Analysis

Data on antibiosis, extracellular enzymatic activity, and antibiotic sensitivity were recorded in excel sheets. The general linear model (PROC GLM) procedure of SAS software version 9.1 (SAS Institute, Cary, NC) was used to perform analysis of variance (ANOVA). Tukey's honest significant difference (HSD) test was used to compare and separate the means of the diameter of zones of inhibition. Correlation profiles of zones of clearance on different substrates and zones of inhibition were visualized as a heatmap generated by a hierarchical clustering *R* script using *R* version 3.3.1 software. The genetic affiliation of the sequenced genes of the isolates was deduced from a phylogenetic tree generated using MEGA X. The analysis involved 20 nucleotide sequences. The evolutionary distances were computed using the maximum composite likelihood method and were recorded as units of the number of base substitutions.

## 3. Results

### 3.1. Effect of Antibiotics on Bacteria Isolated from Food and Soil

**Figure 2:**
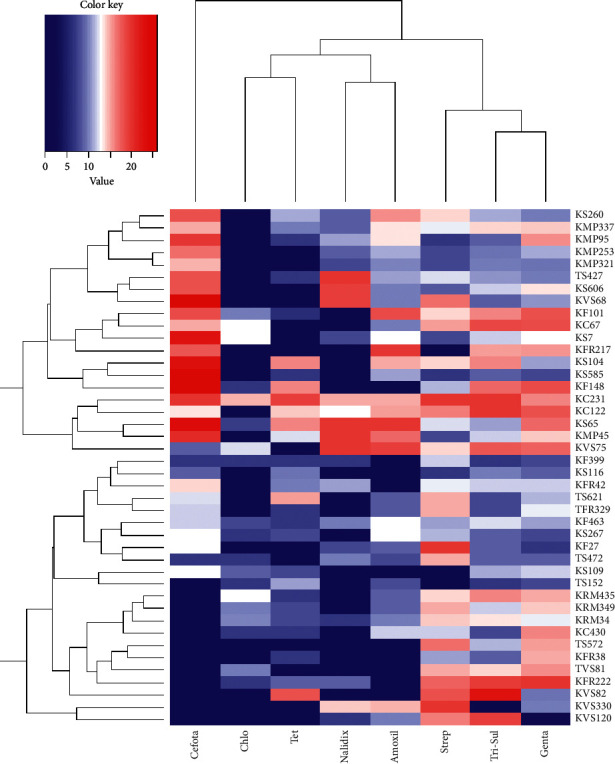
Activity of eight antibiotics against antibiotic-resistant bacteria isolated from vended food and soil in Embu Town and Kangaru Market. Strep, streptomycin; Genta, gentamycin; Amox, amoxicillin; tetra, tetracycline; Chlo, chloramphenicol; Tri-Sul, trimethoprim + sulphamethoxazole; Cefotax, cefotaxime; Nalid, nalidixic acid.

**Figure 3:**
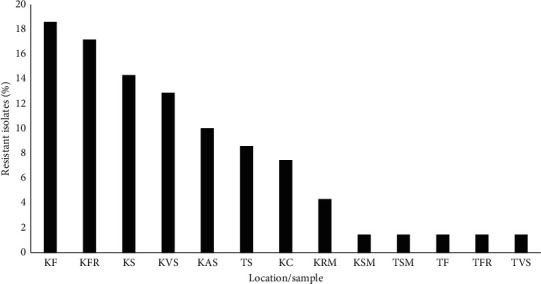
Proportion of antibiotic-resistant bacteria from food vended in Embu Town and Kangaru Market. KAS, Kangaru African sausage; KRM, Kangaru roasted meat; KF, Kangaru fish; TVS, town vegetable salad; KVS, Kangaru vegetable salad; KC, Kangaru chips; TS, town soil; KSM, Kangaru smokies; KFR, Kangaru fruit; KS, Kangaru soil; TFR, town fruit; TF, town fruits; KAS, Kangaru African sausage.

Antibiotic sensitivity test assays revealed that 70 out of 345 bacterial isolates were resistant to at least three (multiple resistant) antibiotics that were tested. Isolate KMP253 affiliated to *Salmonella* spp., TF152 affiliated to *E. coli*, and KS120 affiliated to *Bacillus subtilis* were resistant to all the antibiotics that were tested and were all isolated from Kangaru Market (Table 2). Hierarchical clustering showed that trimethoprim + sulphamethoxazole was the most effective antibiotic, while chloramphenicol was the least effective antibiotic. Isolate KVS120 was resistant to the highest number of antibiotics (Figure 2). The occurrence of antibiotic-resistant bacteria varied among different food samples. Smokies from both sites had the lowest number of resistant bacterial isolates (1.43%) compared to the other food samples. Fish sold in Kangaru Market had the highest proportion of resistant isolates (18.57 %), as shown in Figure 3. No bacteria were recovered from treated water provided by EWASCO.

### 3.2. Detection of Antibiotic-Resistant Genes

**Figure 4:**
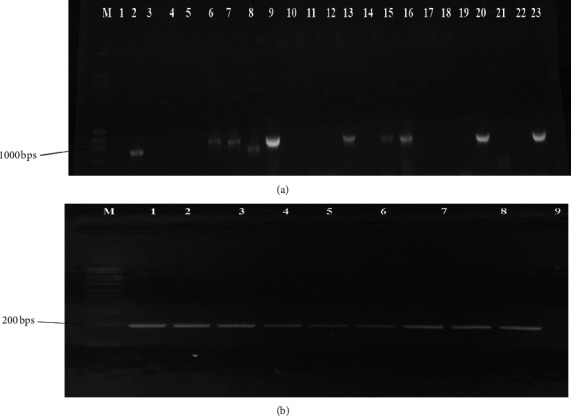
(a) Antibiotic resistance gene ^*Bla*^TEM detected in bacterial isolates that were resistant to beta-lactam antibiotics. *M* is the molecular marker; lane 2, isolate KFR222; 6, KF169; 7, KF463, 8, KMP253; 9, KFR285; 13, KF27; 15, KMP188; 16, KF148; 20, KC67; 23, TS572. The band size is in base pairs. (b) Antibiotic resistance gene *Dfr* A detected in isolates that were resistant to trimethoprim. *M* is the molecular marker; lane 1, isolate KMP95; 2, TFR329; 3, KFR301; 4, KVS330; 5, TS380; 7, TVS81; 8, KF27; 9, KF391; 10, KF391. The band size is in base pairs.

Antibiotic resistance genes were detected in 36 out of the 70 antibiotic-resistant bacterial isolates (Table 3). The antibiotic resistance genes ^*Bla*^TEM and *Dfr* A were frequently detected (Figures 4(a) and 4(b)). Resistance genes were detected at a higher frequency in bacteria isolated from Kangaru Market compared to Embu Town.

### 3.3. Antibiosis Activity

Eleven out of the 70 antibiotic-resistant bacterial isolates inhibited the growth of at least one of the following laboratory strains of bacteria: *S. aureus* ATCC 1026, *E. coli* ATCC 35218, and *K. pneumoniae* 1792880. Isolate KFR38 affiliated to *B. pacificus* inhibited two laboratory strains and exhibited large zones of inhibition (Table 4). None of the antibiotic-resistant isolates was able to inhibit the growth of *K. pyrope* 1792880, *S. pneumoniae* ATCC 49619, or *S. aureus* BAA 976.

### 3.4. Risk Groups of Antibiotic-Resistant Bacterial Isolates

The 14 antibiotic-resistant bacteria that were successfully sequenced belonged to risk groups 1, 2, and 3. Isolate KS606 affiliated to *B. anthracis* and isolate KMP95 affiliated to *S. marcescens* which are risk group 3 organisms that were isolated from food sold in Kangaru Market (Table 5).

### 3.5. Substrate Utilization by Antibiotic-Resistant Bacteria

The antibiotic-resistant bacterial isolates produced different extracellular enzymes, as shown by the zones of clearance on different substrates. Isolates KF148, KF317, KS116, KS7, and KVS249 exhibited the largest zones of clearance with an average diameter and standard error (SE) of 14.67 ± 0.33 mm, 14.00 ± 0.58 mm, 14.00 ± 0.58 mm, 15.00 ± 0.00 mm, and 14.67 ± 0.33 mm, respectively. *B. anthracis* (KS606) recorded the largest zone of clearance on cellulose, while KC430 affiliated to *Pseudomonas* spp. had the largest zone of precipitation on the tween-20 substrate.

### 3.6. Amplification of 16S rRNA Genes and Phylogenetic Analysis

**Figure 5:**
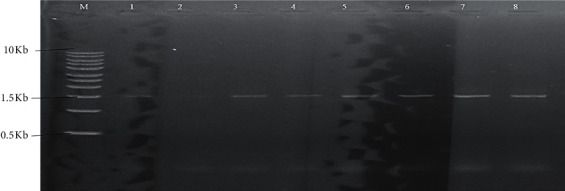
Amplification of bacterial 16S rRNA gene. *M* is the molecular marker (1kb plus ladder); lane 1, isolate KVS68; 2, KMP45; 3, KC75; 4, TS380; 5, KVS214; 6, KFR38; 7, KS376; 8, KMP253.

The 16S rRNA gene was successfully amplified in 47 isolates out of the 70 antibiotic-resistant bacterial isolates (Figure 5).

**Figure 6:**
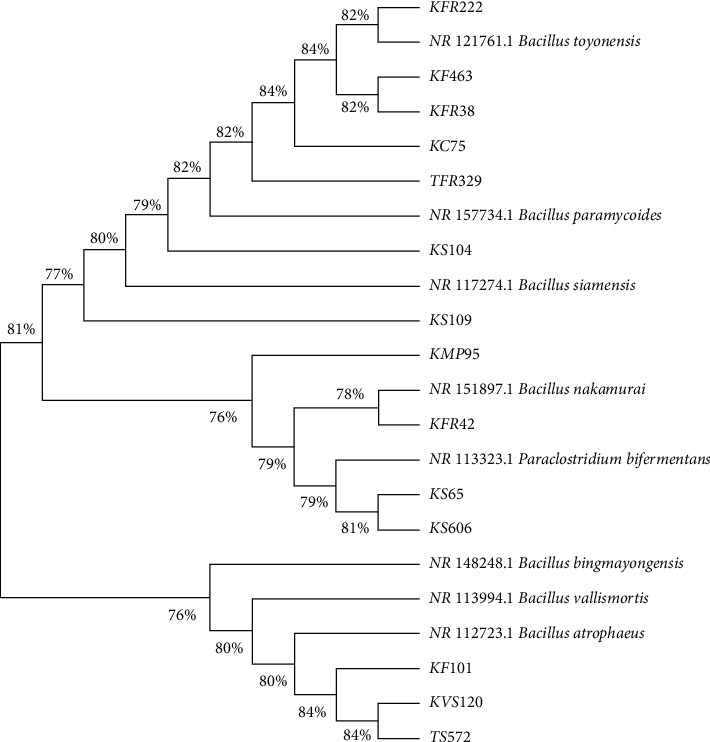
Phylogenetic relationships of bacterial isolates from vended food and soil in Embu Town and Kangaru Market. The evolutionary history was inferred through the neighbor-joining method. Isolates from the present study are presented in the phylogenetic tree using the isolate number codes.

Phylogenetic analysis of successfully sequenced bacterial isolates revealed that the isolates belonged to five genera, namely, *Bacillus*, *Paraclostridium*, *Lysinibacillus*, *Virgibacillus,* and *Serratia* with the similarity of above 90%. The *Bacillus* clade had the highest number of successfully amplified bacterial isolates distributed in nine subclades with bootstrap values of 98 (Figure 6).

## 4. Discussion

The present study isolated 70 bacterial isolates that were resistant to at least three antibiotics from vended foods and the soil in Embu Town and Kangaru Market. Higher levels of antibiotic resistance were recorded from bacteria isolated from vended food and soil in Kangaru Market compared to Embu Town. The presence of antibiotic-resistant pathogens in street vended foods has been recently reported in Kisumu, Kenya [23]. An earlier study in Kibera, Kenya, reported that food and the environment are reservoirs of antibiotic resistance [24]. Food contaminated with antibiotic-resistant bacteria is a threat to public health as it encourages persistence and dissemination of resistance determinants [25]. The higher level of antibiotic resistance recorded in the Kangaru Market may be due to laxity in the enforcement of public health measures. Resistance to gentamycin and streptomycin which are administered by intramuscular injection was 48.57% and 32.86%, respectively. The intramuscular mode of administration possibly limits the use of these antibiotics and thus contributes to the preservation of the antimicrobial activity [26]. In the present study, the frequency of antibiotic resistance to oral antibiotics was high and ranged from 72.86% for amoxicillin to 81.43% for nalidixic acid. The high level of resistance to oral antibiotics is possibly due to availability, convenient mode of administration, broad-spectrum activity, and affordability.

Fish samples in the present study had the highest (18.52%) proportion of antibiotic-resistant isolates. The contamination of fish with antibiotic-resistant bacteria has been previously attributed to fecal contamination of water due to the unhygienic disposal of human waste [27]. Earlier studies reported contamination of water with bacteria that were multidrug resistant to sulphamethoxazole/trimethoprim, ampicillin, and tetracycline [24, 28]. The present study confirmed that treated water from EWASCO that is used by most of the people in Embu Town and Kangaru Market was free from contamination by bacteria at the time of sampling. Therefore, it is unlikely that antibiotic resistance in this study originated from EWASCO water.

The current study isolated antibiotic-resistant bacteria from vegetable salad and African sausages, which are mostly homemade. Contamination of smokies, which are also a kind of sausage that is factory made and packed, with antibiotic-resistant bacteria was low. Postharvest contamination of vegetables and cross-contamination of African sausages are a major risk factor for disseminating antibiotic resistance [29, 30]. Soil samples from both Embu Town and Kangaru Market recorded a higher proportion of antibiotic-resistant isolates compared to food samples. In a previous study, anthropogenic activities such as long-term manure application were shown to increase antibiotic-resistant bacteria in soil [31]. Such activities were not deduced in the present study. Isolation of *Pseudomonas* spp. from both soil and fish in Kangaru Market indicates the possibility of food contamination originating from the environment.

The present study showed that antibiotic resistance was encoded by *Tet*A, ^*Bla*^*TEM*, *StrB*, *Dfr*A, *Amp*, and *FloR* genes. In this study, genes encoding streptomycin resistance *StrB* were detected in only nine out of the 23 isolates that were resistant to streptomycin. The *StrB* genes are known to be encoded by both transposons and plasmids [32]. The present study did not examine transposon-encoded antibiotic resistance, which could explain the detection of *StrB* in fewer isolates. Five of the nine isolates positive for *FloR* genes were isolated from food. This could be due to the use of chloramphenicol for domestic purposes [33]. Contamination of food by antibiotic-resistant bacteria could become a major threat to public health since the antibiotic resistance determinants can be transferred to pathogenic bacteria thus complicating the treatment of bacterial infections.

Sequencing of the 16S rRNA gene revealed that most of the antibiotic-resistant bacterial food contaminants detected in this study belonged to the genus *Bacillus*. Although previous studies have associated *Bacillus* with food spoilage [34], isolation of *B. wiedmannii*, a risk group 2 from food, and *B. anthracis,* a risk group 3 organism from the soil, is of public health concern. The pathogen *B. anthracis* can cause the death of both human beings and animals [35] (https://my.absa.org/tiki-index). Since *S. marcescens* occurs naturally in soil and water, it can cause urinary, respiratory, meningitis, pneumonia, septicemia, and endocarditis infections in man. Isolation of *S. marcescens* from the African sausage in this study could be a result of contamination during preparation, as African sausages are mostly homemade. The presence of *S. marcescens* a risk group 3 organism [7] in food is of grave concern.

Although isolate TS572 affiliated to *B. subtilis* was resistant to five antibiotics, it also had antimicrobial activity against *K. pneumoniae* ATCC 49619. This is not surprising as *B. subtilis* from soil have been reported to produce antimicrobial agents [36]. KMP337 affiliated to *C. freundii* recorded the highest antibiosis activity against *S. aureus* ATCC 1026. Previous studies associated *C. freundii* with multiple antibiotic resistance but not the production of antimicrobial agents [37]. The present study isolated *B. amyloliquefaciens* from fruits in Kangaru Market. In a previous study, *B. amyloliquefaciens* was isolated from banana fruit surfaces and was shown to produce antimicrobial activity against crown root causing pathogens. Thus, *B. amyloliquefaciens* could be explored further for antimicrobial agents [38]. Isolation of bacteria with antimicrobial activity in the present study supports the need for innovative screening strategies in the search for new antimicrobial agents. Most of the bacteria isolated in the current study were able to produce extracellular enzymes. Other studies have suggested that isolates that exhibit high enzymatic activity also possess high antibiosis activity; thus, these processes could be occurring concurrently [18, 39].

## 5. Conclusions

This study showed that vended food and the soil in Embu Town and Kangaru Market contain bacterial food contaminants that are resistant to commonly used antibiotics. The study further confirmed that the bacteria harbored antibiotic resistance determinants *Tet*A, ^*Bla*^*TEM, StrB, Dfr*A*, Amp,* and *FloR.* Some of the bacterial food contaminants isolated in this study are risk group 2 and 3 organisms which is a public health concern. More resistance was recorded in the bacteria originating from Kangaru Market as compared to Embu Town. Overall, vended food in Kangaru Market may pose a health risk to the public if mitigating measures are not put in place.

## Figures and Tables

**Table 1:** List of primers for antibiotic resistance genes.

Primer	Sequence (5′ to 3′)	Target gene	Amplicon size (bp)	Annealing temperature (°C)	Resistance to antibiotics
Flor-F	TTATCTCCCTGTCGTTCCAGCG	*FloR*	450	57.5	Chloramphenicol
Flor-R	CCTATGAGCACACGGGGAGC				

strB-F	GGCACCCATAAGCGTACGCC	*Str* B	400	61	Streptomycin
strB-R	TGCCGAGCACGGCGACTACC				

DfrA1-F	CGAAGAATGGAGTTATCGGG	*Dfr* A	200	55	Trimethoprim
DfrA1-R	TGCTGGGGATTTCAGGAAAG				

TEM-F	GCGGTATCATTGCAGCACTG	^*Bla*^TEM	1000	55	Beta-lactam
TEM-R	TGCTTAATCAGTGAGGCACC				

Amp-F	ATGCACACGCTGATCGGATT	*Amp*	268	65	Ampicillin
Amp-R	GCGGACGCAGACTTCACTAA				

Tet-F	AACCGGCATTGAGAGCATCA	*Tet* A	699	60	Tetracycline
Tet-R	TTGTCTCCTCTCCCTTGGCT				

**Table 2:** Antibiotic resistance of bacteria isolated from vended food and the soil in Embu Town and Kangaru Market in 2018.

Isolate	Probable identity	Location	Sample	Strep	Genta	Amox	Tetra	Chlo	Tri-sul	Cefotax	Nalid	Resistant to antibiotics (*n*)
KS7	*Providencia* spp.	Kangaru	Soil	R	I	R	R	I	I	I	R	4
KF27	*Shigella* spp.	Kangaru	Fish	S	R	R	R	R	R	R	R	7
KRM34	*E. coli*	Kangaru	Roasted meat	S	I	R	R	R	I	R	R	5
KFR38	*Bacillus pacificus*	Kangaru	Fruit	I	S	R	R	R	R	R	R	6
KFR42	*Bacillus amyloliquefaciens*	Kangaru	Fruit	I	R	R	R	R	I	R	R	6
KMP45	*Pseudomonas* spp.	Kangaru	African sausage	R	I	I	I	R	I	R	S	3
KFR52	*Bacillus megaterium*	Kangaru	Fruit	I	I	R	R	R	I	R	R	5
KSM63	*Shigella sonnei*	Kangaru	Smokies	R	S	I	R	I	I	R	I	3
KS65	*Bacillus wiedmannii*	Kangaru	Soil	I	S	R	S	R	I	R	S	3
KC67	*Pseudomonas aeruginosa*	Kangaru	Chips	S	S	R	R	I	S	R	R	4
KVS68	*Enterobacter* spp.	Kangaru	Vegetable salad	S	R	R	R	R	R	S	S	5
KVS75	*Bacillus wiedmannii*	Kangaru	Vegetable salad	I	S	S	R	R	S	R	S	3
TVS81	*Bacillus thuringiensis*	Kangaru	Vegetable salad	S	S	R	R	R	I	R	R	5
KVS82	*Serratia marcescens*	Kangaru	Vegetable salad	S	R	R	S	R	S	R	R	5
KVS85	*Serratia marcescens*	Kangaru	Vegetable salad	S	S	R	S	R	S	I	R	3
KMP95	*Serratia marcescens*	Kangaru	African sausage	R	S	I	R	R	R	R	R	6
KF101	*Bacillus velezensis*	Kangaru	Fish	S	S	S	R	R	S	R	R	4
KS104	*Virgibacillus phasianinus*	Kangaru	Soil	S	R	I	S	R	S	S	R	3
KS109	*Lysinibacillus parviboronicapiens*	Kangaru	Soil	R	R	R	R	R	I	R	R	7
KS116	*Providencia* spp.	Kangaru	Soil	R	R	R	R	R	R	R	R	8
KVS120	*Bacillus subtilis*	Kangaru	Vegetable salad	R	R	R	R	R	R	R	R	8
KC122	*Pseudomonas* spp.	Kangaru	Chips	S	S	I	I	R	S	R	R	3
KFR131	*Pseudomonas* spp.	Kangaru	Fruit	R	I	R	R	R	I	R	R	6
KVS147	*Staphylococcus aureus*	Kangaru	Vegetable salad	R	R	R	S	R	S	S	R	5
KF148	*Shigella* spp.	Kangaru	Fish	I	S	R	S	R	S	S	R	3
TF152	*E. coli*	Town	Fish	R	R	R	R	R	R	R	R	8
KMP159	*Salmonella* spp.	Kangaru	African sausage	I	R	I	S	R	S	S	R	3
KS160	*Citrobacter* spp.	Kangaru	Soil	R	R	R	R	R	I	R	R	7
KF169	*Proteus* spp.	Kangaru	Fish	R	R	R	S	R	S	S	R	5
KMP188	*Aerobacter aerogenes*	Kangaru	African sausage	R	R	R	S	R	S	S	R	5
KFR200	*Enterobacter* spp.	Kangaru	Fruit	S	I	S	R	R	I	R	I	3
KFR204	*Pseudomonas* spp.	Kangaru	Fruit	S	I	R	R	R	I	R	R	5
KVS214	*S. pneumoniae*	Kangaru	Vegetable salad	R	R	R	R	S	R	R	R	7
KFR217	*Klebsiella* spp.	Kangaru	Fruit	R	S	S	R	R	S	R	R	5
KFR222	*Bacillus mobilis*	Kangaru	Fruit	S	S	R	R	R	S	R	R	5
KC231	*S. pneumoniae*	Kangaru	Chips	S	S	I	S	R	S	R	R	3
KFR245	*P. aeruginosa*	Kangaru	Fruit	S	S	I	I	R	I	R	R	3
KF246	*S. pneumoniae*	Kangaru	Fish	I	S	I	R	R	I	R	R	4
KVS249	*Pseudomonas* spp.	Kangaru	Vegetable salad	I	R	R	R	R	R	R	R	7
KMP253	*Salmonella* spp.	Kangaru	African sausage	R	R	R	R	R	R	R	R	8
KS260	*E. coli*	Kangaru	Soil	I	R	S	R	R	I	R	R	5
KS267	*Bacillus wiedmannii*	Kangaru	Soil	I	R	R	R	R	R	R	R	7
KVS271	*Pseudomonas* spp.	Kangaru	Vegetable salad	I	R	I	R	R	I	R	R	5
KFR285	*Hafnia* spp.	Kangaru	Fruit	S	R	I	S	R	S	S	R	3
KFR286	*Pseudomonas* spp.	Kangaru	Fruit	I	S	R	R	R	S	R	R	5
KFR301	*Salmonella* spp.	Kangaru	Fruit	I	R	R	R	R	R	R	R	7
KF317	*Streptococcus pneumoniae*	Kangaru	Fish	R	S	I	R	R	I	R	R	5
KMP321	*Bacillus wiedmannii*	Kangaru	African sausage	R	R	R	R	R	I	R	R	7
TFR329	*Bacillus proteolyticus*	Town	Fruit	S	R	R	R	R	R	R	R	7
KVS330	*Citrobacter freundii*	Kangaru	Vegetable salad	S	R	I	R	R	R	R	I	5
KMP337	*Salmonella* spp.	Kangaru	African sausage	I	I	I	R	R	I	R	R	4
KRM349	*Pseudomonas* spp.	Kangaru	Roasted meat	I	I	R	R	R	I	R	R	5
KS376	*E. coli*	Kangaru	Soil	S	R	R	R	R	R	R	R	7
TS378	*Salmonella* spp.	Town	Soil	I	R	R	R	R	I	R	R	6
TS380	*Pseudomonas* spp.	Town	Soil	R	S	R	R	R	R	R	R	7
KF381	*Micrococcus* spp.	Kangaru	Fish	S	R	R	R	R	R	R	R	7
KF389	*Morganella* spp.	Kangaru	Fish	I	S	R	I	R	I	R	S	3
KF391	*Serratia marcescens*	Kangaru	Fish	R	R	R	R	R	R	R	S	7
KF393	*Yersinia* spp.	Kangaru	Fish	S	R	R	R	R	I	R	R	6
KF395	*Pseudomonas aeruginosa*	Kangaru	Fish	S	I	R	R	R	S	R	R	5
KF399	*Salmonella enterica*	Kangaru	Fish	I	R	R	R	R	R	R	R	7
TS427	*Pseudomonas* spp.	Town	Soil	I	R	R	R	R	I	R	S	5
KC430	*Pseudomonas* spp.	Kangaru	Chips	I	S	R	R	R	R	R	R	6
KRM435	*Bacillus wiedmannii*	Kangaru	Roasted meat	I	S	R	R	I	S	R	R	4
KF463	*Bacillus anthracis*	Kangaru	Fish	R	R	R	R	R	I	R	R	7
TS472	*Bacillus cereus*	Town	Soil	S	R	R	R	R	R	R	R	7
TS572	*Bacillus subtilis*	Town	Soil	S	S	R	R	R	I	R	R	5
KS585	*Enterococcus faecalis*	Kangaru	Soil	R	R	R	R	R	R	S	R	7
KS606	*Bacillus anthracis*	Kangaru	Soil	R	I	R	R	R	I	R	S	5
TS621	*Proteus* spp.	Town	Soil	S	R	R	S	R	I	R	R	5

Strep, streptomycin; Genta, gentamycin; Amox, amoxicillin; tetra, tetracycline; Chlo, chloramphenicol; Tri-Sul, trimethoprim + sulphamethoxazole; Cefotax, cefotaxime; Nalid, nalidixic acid.

**Table 3:** Detection of antibiotic resistance genes in bacteria isolated from vended food and soil in Embu Town and Kangaru Market.

Isolate number	Location	Sample	Probable identity	Detected antibiotic resistance genes					
				*Dfr* A	*Str* B	*Tet* A	^*Bla*^TEM	*Amp*	*FloR*
KFR222	Kangaru	Fruit	*B. mobilis*	−	−	−	+	−	−
KF169	Kangaru	Fish	*Proteus* spp.	−	−	−	+	−	−
KF463	Kangaru	Fish	*B. anthracis*	−	+	−	+	−	−
KMP253	Kangaru	African sausage	*Salmonella* spp.	−	+	−	+	−	−
KFR285	Kangaru	Fruit	*Hafnia* spp.	−	−	+	+	−	−
KF27	Kangaru	Fish	*Shigella* spp.	−	−	−	+	−	+
KMP188	Kangaru	African sausage	*A. aerogenes*	−	−	−	+	+	−
KF148	Kangaru	Fish	*Shigella* spp.	−	−	−	+	−	+
KC67	Kangaru	Chips	*P. aeruginosa*	−	−	−	+	−	−
TS572	Town	Soil	*B. subtilis*	−	+	−	+	−	+
KMP95	Kangaru	African sausage	*S. marcescens*	+	−	−	−	−	+
TFR329	Town	Fruit	*B. proteolyticus*	+	−	+	−	−	−
KFR301	Kangaru	Fruit	*Salmonella* spp.	+	−	−	−	+	−
KVS330	Kangaru	Vegetable salad	*E. coli*	+	−	+	−	−	−
TS380	Town	Soil	*Salmonella* spp.	+	−	−	−	−	−
TVS81	Town	Vegetable salad	*B. thuringiensis*	+	−	−	−	+	−
KF391	Kangaru	Fish	*Morganella* spp.	+	+	+	−	−	−
KS160	Kangaru	Soil	*Citrobacter* spp.	−	+	−	−	−	−
KF169	Kangaru	Fish	*Proteus* spp.	−	+	−	−	+	−
KF317	Kangaru	Fish	*E. faecalis*	−	+	−	−	−	−
KFR131	Kangaru	Fruit	*Pseudomonas* spp.	−	+	−	−	−	−
KFR38	Kangaru	Fruit	*B. pacificus*	−	+	−	−	−	−
KVS85	Kangaru	Vegetable salad	*S. marcescens*	−	−	−	−	+	−
KFR204	Kangaru	Fruit	*Pseudomonas* spp.	−	−	−	−	+	−
KS109	Kangaru	Soil	*L. parviboronicapiens*	−	−	−	−	+	−
KVS214	Kangaru	Vegetable salad	*S. pneumoniae*	−	−	−	−	+	−
KS585	Kangaru	Soil	*B. toyonensis*	−	−	−	−	+	+
KF399	Kangaru	Fish	*P. aeruginosa*	−	−	+	−	+	−
KS267	Kangaru	Soil	*B. wiedmannii*	−	−	−	−	+	−
KS116	Kangaru	Soil	*Providencia* spp.	−	−	−	−	+	−
KC122	Kangaru	Chips	*Pseudomonas* spp.	−	−	−	−	−	+
KFR245	Kangaru	Fruit	*P. aeruginosa*	−	−	−	−	−	+
KS376	Kangaru	Soil	*Pseudomonas* spp.	−	−	−	−	−	+
KVS249	Kangaru	Vegetable salad	*Pseudomonas* spp.	−	−	+	−	−	−
KS606	Kangaru	Soil	*B. anthracis*	−	−	+	−	−	−
KF393	Kangaru	Fish	*S. marcescens*	−	−	+	−	−	−

+ indicates the presence of the resistance genes following amplification by PCR; − indicates the absence of the target resistance genes following amplification by PCR.

**Table 4:** Antibiosis effect of antibiotic-resistant bacteria isolated from vended food and soil on laboratory strains of bacteria.

Isolate Number	Probable identity	Location	Sample	*E. coli* ATCC 35218	*S. aureus* ATCC 1026	*K. pneumoniae* ATCC 35218
KC430	*Pseudomonas* spp.	Kangaru	Chips	0.00 ± 0.00^b^	0.00 ± 0.00^b^	14.33 ± 0.33^a^
KC67	*P. aeruginosa*	Kangaru	Chips	0.00 ± 0.00^b^	0.00 ± 0.00^b^	10.00 ± 0.00^b^
KF395	*Yersinia* spp.	Kangaru	Fish	0.00 ± 0.00^b^	0.00 ± 0.00^b^	9.67 ± 0.33^b^
KFR217	*Klebsiella* spp.	Kangaru	Fruit	0.00 ± 0.00^b^	0.00 ± 0.00^b^	7.00 ± 0.00^c^
KFR38	*B. pacificus*	Kangaru	Fruit	0.00 ± 0.00^b^	8.33 ± 2.33^b^	13.67 ± 0.33^a^
KMP337	*C. freundii*	Kangaru	Meat pie	0.00 ± 0.00^b^	16.33 ± 0.88^a^	0.00 ± 0.00^d^
KS260	*E. coli*	Kangaru	Soil	7.00 ± 0.00^a^	0.00 ± 0.00^b^	9.00 ± 0.00b^c^
KSM63	*S. sonnei*	Kangaru	Samosa	0.00 ± 0.00^b^	0.00 ± 0.00^b^	10.33 ± 1.33^b^
KVS271	*Pseudomonas* spp.	Kangaru	Vegetable salad	0.00 ± 0.00^b^	0.00 ± 0.00^b^	7.00 ± 0.00^c^
TS380	*Salmonella* spp.	Town	Soil	0.00 ± 0.00^b^	0.00 ± 0.00^b^	11.00 ± 0.58^b^
TS572	*B. subtilis*	Town	Soil	0.00 ± 0.00^b^	0.00 ± 0.00^b^	14.00 ± 0.58^a^

*P* value				0.001	0.001	0.001
CV				0.00	76.76	9.03
LSD				0.00	3.80	2.54

Mean with SE for antibiosis activity of the isolates, where the means with the same letters are not significantly different as indicated by Tukey's HSD test (*P* < 0.05).

**Table 5:** Risk groups of antibiotic-resistant bacteria isolated from vended foods and soil in Embu Town and Kangaru Market.

Isolate no.	Location	Sample	Identity	% similarity	Resistant to antibiotics (*n*)	Resistance genes detected by PCR	Riskgroup
KFR42	Kangaru	Fruit	*B. amyloliquefaciens*	99.44	6	—	1
KFR38	Kangaru	Fruit	*B. pacificus*	99.1	6	*Str*B	1
KS65	Kangaru	Soil	*P. benzoelyticum*	99.57	3	—	1
KC75	Kangaru	Chips	*B. wiedmannii*	99.51	3	—	2
KF101	Kangaru	Fish	*B. velezensis*	97.86	4	—	1
KVS120	Kangaru	Vegetable salad	*B. subtilis*	97.94	8	—	1
KFR222	Kangaru	Fruit	*B. mobilis*	99.83	5	^*Bla*^TEM	2
KF463	Kangaru	Fish	*B. anthracis*	99.43	7	*Str*B, ^*Bla*^TEM	1
TFR329	Town	Fruit	*B. proteolyticus*	97.18	7	*Str* B, *Dfr* A	1
KS109	Kangaru	Soil	*L. parviboronicapiens*	90.83	7	*Amp*	1
KS104	Kangaru	Soil	*V. phasianinus*	99.31	3	—	1
KMP95	Kangaru	African sausage	*S. marcescens*	95.39	6	*Dfr* A, *FloR*	3
KS606	Kangaru	Soil	*B. anthracis*	100	5	*Tet* A	3
TS572	Town	Soil	*B. subtilis*	97.51	5	*Str* B, ^*Bla*^TEM, *FloR*	1

– ,resistance gene not detected.

## Data Availability

Additional data for this manuscript have been provided as supplementary material.

## Supplementary Materials

Supplementary Materials Supplementary Table 1: morphological and biochemical characterization of antibiotic-resistant bacteria isolated from Embu Town and Kangaru Market in 2018. Supplementary Table 2: drug sensitivity interpretation of zone of inhibition diameter based on CLSI (Standard and Testing Guidelines 2018). Supplementary Table 3: enzymatic activity of the bacteria isolated from vended food and soil in Embu Town and Kangaru Market. Supplementary Figure 1: enzymatic activity of the antibiotic-resistant bacterial isolates isolated from fast food and the soil in Embu Town and Kangaru Market.
