# Supplementary material for: Detection and Profiling of Antibiotic Resistance among Culturable Bacterial Isolates in Vended Food and Soil Samples
Source: Int J Microbiol. 2020 Sep 4;2020:6572693. doi: 10.1155/2020/6572693 (PMC7493785; doi:10.1155/2020/6572693)
Supplement: Supplementary Materials — Supplementary Table 1: morphological and biochemical characterization of antibiotic-resistant bacteria isolated from Embu Town and Kangaru Market in 2018. Supplementary Table 2: drug sensitivity interpretation of zone of inhibition diameter based on CLSI (Standard and Testing Guidelines 2018). Supplementary Table 3: enzymatic activity of the bacteria isolated from vended food and soil in Embu Town and Kangaru Market. Supplementary Figure 1: enzymatic activity of the antibiotic-resistant bacterial isolates isolated from fast food and the soil in Embu Town and Kangaru Market. [file 6572693.f1.docx]

**Supplementary material**

Supplementary Table 1: Morphological and biochemical characterization of antibiotic resistant bacteria isolated from Embu Town and Kangaru Market in 2018

| Isolate | Lactose | Sucrose | Glucose | Deamination | Catalase | Citrate | H2S | Cracks | Cell shape | Gram | Probable Identity |
| --- | --- | --- | --- | --- | --- | --- | --- | --- | --- | --- | --- |
| KS7 | - | - | + | - | ++ | + | - | + | Rod | - | *Providencia* spp., |
| KF27 | - | - | - | - | +++ | - | - | - | Rod | - | *Shigella* spp., |
| KRM34 | + | + | + | - | +++ | - | - | - | Rod | - | *E.coli* |
| KFR38 | - | - | - | - | + | - | - | - | Rod | + | *Bacillus pacificus* |
| KFR42 | - | - | - |  | +++ | + | - | - | Rod | + | *Bacillus amyloliquefaciens* |
| KMP45 | + | + | + | - | +++ | + | - | - | Rod | - | *Pseudomonas* spp., |
| KFR52 | - | - | + | - | +++ | + | - | - | Rod | - | *Bacillus megaterium* |
| KSM63 | + | + | + | - | - | - | - | - | Rod | - | *Shigella sonnei* |
| KS65 | - | - | + | - | +++ | + | - | - | Rod | + | *Paraclostridium benzoelyticum* |
| KC67 | - | - | + | - | ++ | - | - | - | Rod | - | *Pseudomonas aeruginosa* |
| KVS68 | - | - | + | - | +++ | - | - | - | Rod | - | *Enterobacter* spp., |
| KC75 | + | + | + | - | - | - | - | - | Rod | - | *Bacillus wiedmannii* |
| TVS81 | - | - | - | - | +++ | - | - | - | Rod | - | *Bacillus thuringiensis* |
| KVS82 | - | - | - | - | +++ | - | - | - | Rod | - | *Serratia marcescens* |
| KVS85 | - | - | - | - | +++ | - | - | - | Rod | - | *Serratia marcescens* |
| KMP95 | + | + | + | - | ++ | + | - | - | Rod | - | *Serratia marcescens* |
| KF101 | - | - | - | - | + | + | - | - | Rod | + | *Bacillus velezensis* |
| KS104 | + | + | + | - | +++ | + | - | + | Rod | + | *Virgibacillus phasianinus* |
| KS109 | + | + | + | - | ++ | - | - | + | Rod | + | *Lysinibacillus parviboronicapiens* |
| KS116 | - | - | + | + | ++ | + | - | - | Rod | - | *Providencia* spp., |
| KVS120 | - | - | - | - | +++ | - | - | - | Rod | + | *Bacillus subtilis* |
| KC122 | - | - | + | - | +++ | - | - | - | Rod | - | *Pseudomonas* spp., |
| KFR131 | - | - | + | - | + | - | - | - | Rod | - | *Pseudomonas* spp., |
| KVS147 | + | + | + | - | ++ | + | - | - | Streptococcus | + | *Staphylococcus aureus* |
| KF148 | + | + | + | - | +++ | - | - | - | Rod | - | *Shigella* spp., |
| TF152 | + | + | + | - | +++ | + | - | - | Rod | - | *E. coli* |
| KMP159 | - | + | + | - | ++ | - | - | - | Rod | - | *Salmonella* spp., |
| KS160 | + | + | + | - | ++ | - | - | - | Long rod | _-_ | *Citrobacter* spp*.,* |
| KF169 | - | - | + | + | ++ | + | - | - | Rod | - | *Proteus spp.,* |
| KMP188 | - | - | + | - | +++ | + | - | - | Short rod | - | *Aerobacter aerogenes* |
| KFR200 | - | - | - | - | ++ | - | - | - | Short rod | - | *Enterobacter* spp., |
| KFR204 | + | + | + | - | +++ | - | - | - | Rod | - | *Pseudomonas* spp., |
| KVS214 | + | + | + | - | +++ | - | - | - | Coccus | + | *S. pneumoniae* |
| KFR217 | + | + | + | - | +++ | - | - | - | Rod | - | *Klebsiella* spp., |
| KFR222 | - | + | + | - | ++ | + | - | - | Rod | + | *Bacillus subtilis* |
| KC231 | - | - | - | - | + | - | - | - | coccus | + | *S. pneumoniae* |
| KFR245 | - | - | - | - | +++ | - | - | - | Coccus | + | *P. aeruginosa* |
| KF246 | - | - | - | - | - | - | - | - | Coccus | + | *S. pneumoniae* |
| KVS249 | - | - | - | - | +++ | + | - | - | short rod | + | *Pseudomonas* spp., |
| KMP253 | - | - | + | - | ++ | - | + | - | Rod | - | *Salmonella* spp |
| KS260 | + | + | + | - | ++ | + | - | + | Rod | - | *E. coli* |
| KS267 | + | + | + | - | ++ | - | - | + | Rod | + | *Bacillus weidmannii* |
| KVS271 | - | - | - | - | + | - | - | - | Rod | - | *Pseudomonas* spp., |
| KFR285 | - | - | - | - | +++ | - | - | - | Rod | - | *Hafnia* spp., |
| KFR286 | - | - | - | - | +++ | - | - | - | Rod | - | *Pseudomonas* spp., |
| KFR301 | - | + | + | - | ++ | - | + | - | Rod | - | *Salmonella* spp., |
| KMP321 | + | + | + | - | +++ | + | - | - | Coccus | + | *Streptococcus pneumoniae* |
| TFR329 | - | - | - | - | + | - | - | - | Rod | + | *Bacillus proteolyticus* |
| KVS330 | + | + | + | - | - | - | - | - | Rod | - | *E. coli* |
| KMP337 | - | - | - | - | +++ | - | - | - | Long rod | - | *Citrobacter freundii* |
| KRM349 | - | + | + | - | - | + | + | - | Rod | - | *Salmonella* spp., |
| KS376 | + | + | + | - | +++ | + | - | + | Rod | - | *Pseudomonas* spp., |
| TS378 | + | + | + | - | ++ | - | - | - | Rod | - | *E.coli* |
| TS380 | + | + | + | - | +++ | + | + | + | Rod | - | *Salmonella* spp., |
| KF381 | - | + | + | - | ++ | - | - | - | Rod | - | *Pseudomonas* spp., |
| KF389 | - | - | + | - | +++ | - | - | - | Coccus | - | *Micrococcus spp.,* |
| KF391 | + | + | + | + | +++ | + | - | - | Rod | - | *Morganella* spp., |
| KF393 | + | + | + | - | ++ | - | - | - | Rod | - | *Serratia marcescens* |
| KF395 | - | - | - | - | +++ | - | - | - | Rod | - | *Yersinia* spp., |
| KF399 | - | - | + | - | + | - | - | - | Rod | - | *Pseudomonas aeruginosa* |
| TS427 | + | + | + | - | +++ | + | - | - | Rod | - | *Salmonella enterica* |
| KC430 | + | + | + | - | +++ | + | - | - | Rod | + | *Pseudomonas* spp., |
| KRM435 | + | + | + | - | +++ | - | - | - | Rod | - | *Pseudomonas* spp., |
| KF463 | + | + | + | - | ++ | - | - | - | Rod | + | Bacillus parathrancis |
| TS572 | - | - | + | - | ++ | + | - | - | Rod | - | *Bacillus subtillis* |
| KS606 | + | + | + | - | + | - | - | + | Rod | + | *Bacillus anthracis* |
| TS621 | + | + | + | + | - | - | - | - | Rod | + | *Bacillus cereus* |
| KF317 | + | + | + | - | ++ | + | - | - | Coccus | + | *Enterococcus feacalis* |
| KS585 | + | + | + | - | - | - | - | - | Rod | + | *Bacillus toyonensis* |
| TS472 | + | + | - | - | +++ | - | - | - | Rod | - | *Proteus* spp., |

Key; + (positive), - (negative), catalase test + (slow), ++ (moderate), +++(vigorous), -(negative)

Supplementary Table 2: Drug sensitivity interpretation of zone of inhibition diameter based on CLSI (Standard and Testing Guidelines 2018)

| Antibiotics | Potency | Susceptible | Intermediate | Resistant |
| --- | --- | --- | --- | --- |
| Amoxicillin | 20 µg | ≥18 | 14-16 | ≤8 |
| Cefotaxime | 30 µg | ≥26 | 23-25 | ≤22 |
| Gentamycin | 10 µg | ≥15 | 13-14 | ≤12 |
| Streptomycin | 10 µg | ≥15 | 13-14 | ≤12 |
| Tetracycline | 30 µg | ≥15 | 12-14 | ≤11 |
| Nalidixic | 30 µg | ≥19 | 14-18 | ≤13 |
| Trimethoprim+sulphamethoxazole | (1.25+23.75 µg | ≥16 | 11-15 | ≤10 |
| Chloramphenicol | 30 µg | ≥18 | 13-17 | ≤12 |

Supplemenray Table 3: Enzymatic activity of bacteria isolated from from vended food and soil in Embu Town and Kangaru Market

| Treatment | Protease | Amylase | Cellulase | Esterase |
| --- | --- | --- | --- | --- |
| KC122 | 7.33±0.33^mnp^ | 0.00±0.00^e^ | 0.00±0.00^j^ | 0.00±0.00^m^ |
| KC231 | 9.00±0.58^ijklmnp^ | 0.00±0.00^e^ | 0.00±0.00^j^ | 0.00±0.00^m^ |
| KC430 | 7.67±0.67^lmnp^ | 0.00±0.00^e^ | 0.00±0.00^j^ | 10.00±0.00^m^ |
| KC67 | 0.00±0.00^q^ | 0.00±0.00^e^ | 0.00±0.00^j^ | 0.00±0.00^m^ |
| KC75 | 20.67±0.58^cdfghijk^ | 11.00±0.00^a^ | 22.00±0.00^b^ | 0.00±0.00^m^ |
| KF101 | 13.67±0.33^abcd^ | 13.67±0.67^a^ | 0.00±0.00^b^ | 0.00±0.00^m^ |
| KF148 | 11.00±0.33^ab^ | 7.00±0.00^abcde^ | 12.00±0.00^j^ | 11.00±0.00^efghi^ |
| KF169 | 9.00±0.58^ijklmnp^ | 0.00±0.00^e^ | 0.00±0.00^j^ | 15.00±0.67^bc^ |
| KF246 | 8.67±0.67^ijklmnp^ | 10.00±5.00^ab^ | 0.00±0.00^j^ | 8.33±0.33^jk^ |
| KF27 | 10.33±0.33^fghijklmn^ | 0.00±0.00^e^ | 0.00±0.00^j^ | 9.00±0.00^ijk^ |
| KF317 | 14.00±0.58^abcd^ | 7.67±3.84^cd^ | 10.00±0.00^cd^ | 0.00±0.00^m^ |
| KF381 | 12.00±0.58^abcdfghi^ | 7.00±0.00^abcde^ | 9.00±1.00^def^ | 0.00±0.00^m^ |
| KF389 | 10.67±0.33^dfghijklm^ | 0.00±0.00^e^ | 7.00±0.00^hig^ | 0.00±0.00^m^ |
| KF391 | 11.67±0.33^abcdfghij^ | 0.00±0.00^e^ | 0.00±0.00^j^ | 0.00±0.00^m^ |
| KF393 | 10.67±0.33^dfghijklm^ | 0.00±0.00^e^ | 7.00±0.00^hig^ | 7.67±0.67^kl^ |
| KF395 | 9.67±0.67^hijklmn^ | 0.00±0.00^e^ | 0.00±0.00^j^ | 0.00±0.00^m^ |
| KF399 | 9.67±0.33^higklmn^ | 10.00±0.00^ab^ | 9.33±1.33^fghijk^ | 10.00±0.00^fghijk^ |
| KF463 | 2.00±2.00^q^ | 0.00±0.00e | 0.00±0.00^j^ | 10.00±0.00^fghijk^ |
| KFR 131 | 12.67±0.33^abcdfgh^ | 7.00±0.00e | 8.00±0.00^efg^ | 10.00±0.00^fghijk^ |
| KFR200 | 10.67±0.67^dfghijklm^ | 7.00±0.00^abcde^ | 10.00±0.00^cd^ | 0.00±0.00^m^ |
| KFR204 | 11.00±0.58^cdfghijkl^ | 0.00±0.00^e^ | 0.00±0.00^j^ | 0.00±0.00^m^ |
| KFR217 | 10.00±0.58^fghijklmn^ | 0.00±0.00^e^ | 0.00±0.00^j^ | 0.00±0.00^m^ |
| KFR222 | 10.00±0.00^q^ | 0.00±0.00^e^ | 0.00±0.00^j^ | 13.67±1.67^m^ |
| KFR245 | 9.00±0.58^ijklmnp^ | 0.00±0.00^e^ | 7.00±0.00^ghi^ | 12.67±1.20^m^ |
| KFR285 | 8.00±1.00^klmnp^ | 3.67±3.67^bcde^ | 0.00±0.00^j^ | 0.00±0.00^m^ |
| KFR286 | 9.33±0.67^ijklmnp^ | 0.00±0.00^e^ | 0.00±0.00^j^ | 10.00±0.00^fghikk^ |
| KFR301 | 11.33±0.33^bcdfghijk^ | 0.00±0.00^e^ | 0.00±0.00^j^ | 11.00±0.00^efghij^ |
| KFR 38 | 6.00±0.00^p^ | 0.00±0.00^e^ | 0.00±0.00^j^ | 0.00±0.00^m^ |
| KFR42 | 0.00±0.00^q^ | 0.00±0.00^e^ | 0.00±0.00^j^ | 0.00±0.00^m^ |
| KFR52 | 7.00±0.00^np^ | 0.00±0.00^e^ | 6.00±0.00^j^ | 11.00±1.00^efghij^ |
| KMP159 | 11.33±0.88^bcdfghijk^ | 0.00±0.00^e^ | 0.00±0.00^j^ | 11.67±0.88^efghij^ |
| KMP188 | 10.33±0.33^fghijklmn^ | 0.00±0.00^e^ | 0.00±0.00^j^ | 14.67±1.45^cbd^ |
| KMP253 | 8.00±0.00^klmnp^ | 2.33±2.33d^e^ | 0.00±0.00^j^ | 9.33±0.67^hijk^ |
| KMP321 | 13.33±0.67^abcdf^ | 10.33±0.33^ab^ | 11.00±0.00^bc^ | 0.00±0.00^m^ |
| KMP337 | 11.00±1.00^cdfghijkl^ | 0.00±0.33^e^ | 7.00±0.00^ghi^ | 0.00±0.00^m^ |
| KMP45 | 11.33±0.88^bcdfghijk^ | 0.00±0.00^e^ | 0.00±0.00^j^ | 0.00±0.00^m^ |
| KMP95 | 8.67±0.67^ijklmnp^ | 7.00±0.00^abcde^ | 0.00±0.00^j^ | 12.00±0.00^defgh^ |
| KRM34 | 8.00±0.58^klmnp^ | 0.00±0.00^e^ | 0.00±0.00^j^ | 0.00±0.00^m^ |
| KRM349 | 9.33±0.88^hijklmnp^ | 0.00±0.00^e^ | 8.00±0.00^efg^ | 0.00±0.00^m^ |
| KRM435 | 8.00±1.00^klmnp^ | 0.00±0.00^e^ | 0.00±0.00^j^ | 0.00±0.00^m^ |
| KS104 | 7.67±0.33^lmnp^ | 7.00±0.00^abcde^ | 0.00±0.00^j^ | 11.00±0.00^efghij^ |
| KS109 | 7.00±0.00^np^ | 8.00±0.00^abcd^ | 7.00±0.00^hig^ | 0.00±0.00^m^ |
| KS116 | 14.00±0.58^abcd^ | 7.00±0.00^abcde^ | 7.00±0.00^hig^ | 12.33±0.88^cdefg^ |
| KS160 | 9.33±0.88^hijklmnp^ | 0.00±0.00^e^ | 0.00±0.00^j^ | 0.00±0.00^j^ |
| KS260 | 12.67±0.33^abcdfgh^ | 0.00±0.00^e^ | 0.00±0.00^j^ | 10.00±0.00^j^ |
| KS267 | 12.00±0.58^abcdfghi^ | 8.00±4.00^abcd^ | 0.00±0.00^j^ | 20.00±0.00^j^ |
| KS376 | 13.00±0.00^abcdfg^ | 12.00±0.00^a^ | 11.00±0.00^bc^ | 9.00±0.00^ijk^ |
| KS585 | 13.67±0.88^abcd^ | 0.00±0.00^e^ | 0.00±0.00^j^ | 0.00±0.00^m^ |
| KS606 | 8.33±1.33^klmnp^ | 8.00±0.00^abcd^ | 14.00±0.58 | 0.00±0.00^m^ |
| KS65 | 9.67±0.33^ghijklmn^ | 0.00±0.00^e^ | 7.67±0.33^fgh^ | 15.33±0.33^b^ |
| KS7 | 15.00±0.00^a^ | 0.00±0.00^e^ | 14.33±0.33^a^ | 5.00±0.00l |
| KSM63 | 12.00±0.58^abcdfghi^ | 0.00±0.00^e^ | 11.00±0.00^a^ | 0.00±0.00^m^ |
| KVS120 | 0.00±0.00^q^ | 0.00±0.00^e^ | 0.00±0.00^j^ | 12.33±1.20^cdefg^ |
| KVS147 | 12.67±0.33^abcdfgh^ | 9.00±0.00^abcd^ | 8.00±0.00^efg^ | 15.33±1.86^b^ |
| KVS214 | 9.67±0.33^ghijklmn^ | 0.00±0.00^e^ | 0.00±0.00^j^ | 0.00±0.00^m^ |
| KVS249 | 14.67±0.33^ab^ | 9.67±2.67^abc^ | 7.00±0.00^ghi^ | 9.67±0.67^ghijk^ |
| KVS271 | 12.67±0.33^abcdfgh^ | 11.33±0.33^a^ | 0.00±0.00^j^ | 0.00±0.00^m^ |
| KVS330 | 10.67±0.33^dfghijklm^ | 0.00±0.00^e^ | 0.00±0.00^j^ | 0.00±0.00^m^ |
| KVS68 | 10.00±0.58^ghijklmn^ | 0.00±0.00^e^ | 0.00±0.00^j^ | 0.00±0.00^m^ |
| KVS82 | 8.33±0.33^jklmnp^ | 7.00±0.00^abcde^ | 0.00±0.00^j^ | 0.00±0.00^m^ |
| KVS85 | 14.33±0.88^abc^ | 0.00±0.00^e^ | 11.00±0.00^bc^ | 0.00±0.00^m^ |
| TF152 | 9.33±0.67^hijklmnp^ | 0.00±0.00^e^ | 0.00±0.00^j^ | 0.00±0.00^m^ |
| TFR329 | 13.00±0.58^abcdfg^ | 3.33±3.33^bcde^ | 0.00±0.00^j^ | 0.00±0.00^m^ |
| TS378 | 10.33±0.33^fghijklmn^ | 0.00±0.00^e^ | 6.33±0.33^hi^ | 9.00±0.00^ijk^ |
| TS380 | 8.33±0.33^jklmnp^ | 0.00±0.00^e^ | 0.00±0.00^j^ | 0.00±0.00^m^ |
| TS427 | 0.00±0.00^q^ | 0.00±0.00^e^ | 0.00±0.00^j^ | 0.00±0.00^m^ |
| TS472 | 10.67±0.33^dfghijklm^ | 2.67±2.67^cde^ | 0.00±0.00^j^ | 0.00±0.00^m^ |
| TS572 | 12.67±0.33^abcdfgh^ | 0.00±0.00^e^ | 0.00±0.00^j^ | 0.00±0.00^m^ |
| TS621 | 8.67±0.33^ijklmnp^ | 10.00±0.00^ab^ | 0.00±0.00^j^ | 0.00±0.00^m^ |
| TVS81 | 6.67±0.33^p^ | 0.00±0.00^e^ | 0.00±0.00^j^ | 0.00±0.00^m^ |
| P value  LSD  CV | 0.001  3.56  10.67 | 0.001  7.21  70.35 | 0.001  1.33  11.81 | 0.001  2.85  17.64 |

Mean with standard error (SE) extracellular activity of antibiotic resistant bacteria isolated from fast food and the surrounding environment in Embu town and nearby Kangaru Market, where means with the same letters are not significantly different as indicated by Turkey’s HSD test (P ≤ 0.05)


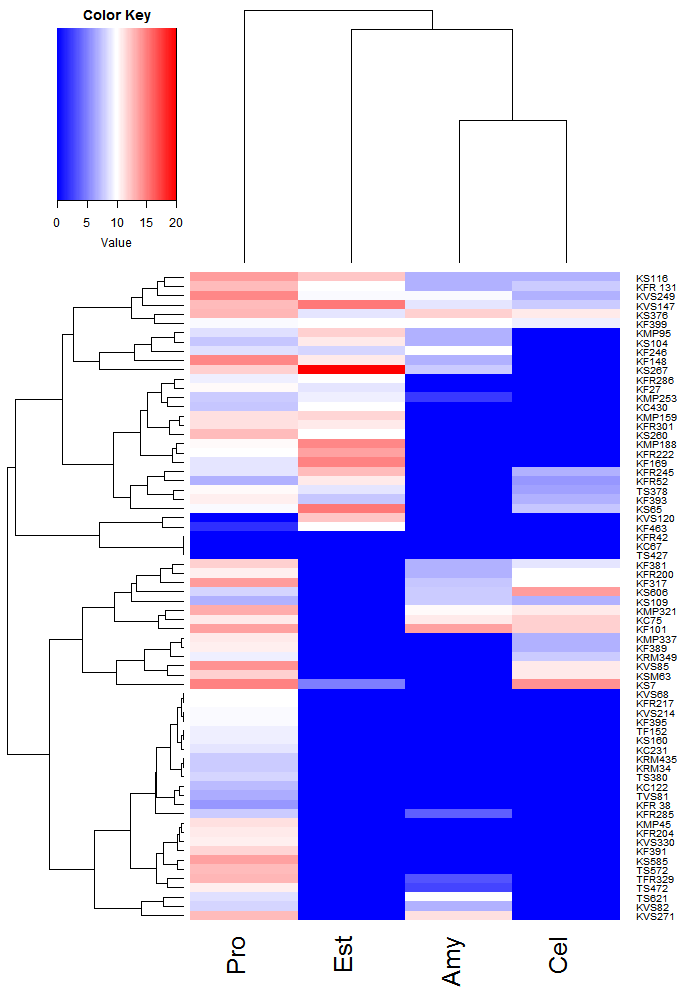


Supplementary Figure 1: Enzymatic activity of the antibiotic resistant bacterial isolates isolated from fast food and the surrounding environment in Embu Town and Kangaru Market

Hierarchical clustergram generated using means of zones of clearance. The heatmap (based on Manhattan metric), shows relationship between selected bacterial isolates and the measured morphometric descriptors (zone of clearance). The colored scale bar represents the quantified significant strength of the assayed morphometric descriptor.
